# Intron-Encoded Domain of Herstatin, An Autoinhibitor of Human Epidermal Growth Factor Receptors, Is Intrinsically Disordered

**DOI:** 10.3389/fmolb.2022.862910

**Published:** 2022-05-02

**Authors:** Daisuke Tashiro, Shunji Suetaka, Nao Sato, Koji Ooka, Tomoko Kunihara, Hisashi Kudo, Junichi Inatomi, Yuuki Hayashi, Munehito Arai

**Affiliations:** ^1^ Department of Life Sciences, Graduate School of Arts and Sciences, The University of Tokyo, Tokyo, Japan; ^2^ Department of Physics, Graduate School of Science, The University of Tokyo, Tokyo, Japan

**Keywords:** human epidermal growth factor receptor, herstatin, intron-encoded protein, intrinsically disordered protein, pre-molten globule state, small-angle X-ray scattering

## Abstract

Human epidermal growth factor receptors (HER/ERBB) form dimers that promote cell proliferation, migration, and differentiation, but overexpression of HER proteins results in cancer. Consequently, inhibitors of HER dimerization may function as effective antitumor drugs. An alternatively spliced variant of HER2, called herstatin, is an autoinhibitor of HER proteins, and the intron 8-encoded 79-residue domain of herstatin, called Int8, binds HER family receptors even in isolation. However, the structure of Int8 remains poorly understood. Here, we revealed by circular dichroism, NMR, small-angle X-ray scattering, and structure prediction that isolated Int8 is largely disordered but has a residual helical structure. The radius of gyration of Int8 was almost the same as that of fully unfolded states, although the conformational ensemble of Int8 was less flexible than random coils. These results demonstrate that Int8 is intrinsically disordered. Thus, Int8 is an interesting example of an intrinsically disordered region with tumor-suppressive activity encoded by an intron. Furthermore, we show that the R371I mutant of Int8, which is defective in binding to HER2, is prone to aggregation, providing a rationale for the loss of function.

## 1 Introduction

**FIGURE 1:**
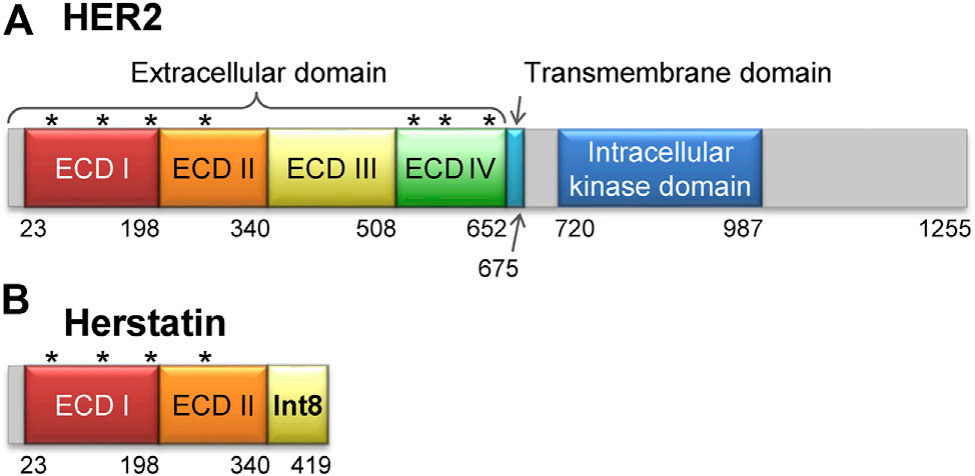
Structures of HER2 and herstatin. **(A)** Structure of HER2. The extracellular domain is composed of four subdomains, ECD I, ECD II, ECD III, and ECD IV. Seven glycosylation sites are shown by asterisks [N68, N124, N187, N259, N530, N571, and N629 according to the UniProt database (Accession number P04626) (UniProt Consortium, 2021)]. **(B)** Structure of herstatin. ECD I and ECD II are identical to those in HER2. An intron-8 encoded 79-residue domain, named Int8 (or ECD IIIa), is retained at the C-terminal region of herstatin. Four glycosylation sites identical to those in HER2 are shown by asterisks. Note that Int8 does not have a glycosylation site.

Human epidermal growth factor receptors (HER/ERBB) are receptor tyrosine kinases that play crucial roles in the regulation of cell proliferation, migration, and differentiation (Citri and Yarden, 2006). The HER family comprises four members, namely HER1 (EGFR), HER2 (NEU), HER3, and HER4 with differential tissue expression patterns. All four members of the family share a common three-dimensional structure that comprises an extracellular domain (ECD), a transmembrane domain, and an intracellular kinase domain (Figure 1A) (Yarden and Pines, 2012). Homo- or hetero-dimerization of the ECD induces autophosphorylation of the intracellular kinase domain, which in turn results in activation of downstream signaling molecules (Normanno et al., 2003; Mujoo et al., 2014). Genetic variants that disrupt the function of these proteins or lead to their overexpression have been associated with multiple cancers (Howe and Brown, 2011). For instance, HER1 variants are associated with lung, breast, and prostate cancers (Normanno et al., 2003), whereas HER2 variants have been found in approximately 30% of all breast cancers (Tai et al., 2010). Thus, the interruption of HER dimerization by specific inhibitors is an effective strategy to halt the growth of cancer cells. Several antibody therapeutics that function *via* this mechanism have been developed, including trastuzumab (herceptin) and pertuzumab that target HER2, and cetuximab that targets HER1.

Herstatin (dimercept) is an alternatively spliced variant of HER2 that retains intron 8 and is secreted by human cells (Doherty et al., 1999; Stix, 2006; Silipo et al., 2017; Hart et al., 2020). It lacks the intracellular kinase and transmembrane domains, but retains a part of the extracellular domain that binds to the ECDs of intact HER proteins. This results in the suppression of HER autophosphorylation and consequently arrests downstream signal transduction (Doherty et al., 1999; Camenisch et al., 2002; Justman and Clinton, 2002; Jhabvala-Romero et al., 2003; Hu et al., 2006; Gompels et al., 2011). Thus, herstatin can autoinhibit the function of HER proteins by binding them and interfering with their dimer formation (Doherty et al., 1999; Azios et al., 2001; Shamieh et al., 2004). Furthermore, herstatin binds intracellular HER2 and possibly prevents its translocation from the endoplasmic reticulum to the cell surface (Hu et al., 2006). Given that herstatin is expressed in non-cancerous breast tissue (Koletsa et al., 2008), but is absent in about 75% of breast carcinomas, it may function as a tumor-suppressor. Consequently, its role as a growth regulatory factor during normal development can be possibly exploited in the development of antitumor therapies (Staverosky et al., 2005; Stix, 2006; Koletsa et al., 2008; Jackson et al., 2013).

Herstatin is a 68-kDa soluble protein, comprising two of the four subdomains of the ECD of HER2 (ECD I and ECD II, respectively; residues 1–340), and a separate domain encoded by intron 8, referred to as Int8 (or ECD IIIa; residues 341–419) (Figure 1B). The 79-residue Int8 domain binds with high affinity to HER1, HER2, HER4, and the insulin-like growth factor 1 receptor (Shamieh et al., 2004; Lv et al., 2012). Even short peptides derived from the Int8 domain are known to bind HER2 (Cha et al., 2017). Interestingly, the Int8 domain inhibited the growth of HER2-overexpressing cells, suggesting the tumor-suppressing activity of Int8 (Lv et al., 2012).

Previous studies show that the isolated Int8 domain has a high affinity for HER proteins. This is evident from the fact that whereas a dissociation constant, *K*_d_, was 14 nM for the binding between the full-length herstatin and HER2 (Doherty et al., 1999), the *K*_d_ for Int8 binding to HER1 and HER2 was 78 ± 10 nM and 50 ± 6 nM, respectively, although Int8 does not have the dimerization arm, which is located in the ECD II (Doherty et al., 1999; Shamieh et al., 2004). In accordance with this, Hu et al. demonstrated that deletion of the Int8 domain from herstatin considerably reduced the affinity with HER2 *in vivo*, suggesting that the Int8 domain plays a predominant role in the binding of herstatin to HER2 (Hu et al., 2005). Moreover, Shamieh et al. reported loss of interaction between the isolated Int8 domain and HER2 as a consequence of the R371I mutation in Int8 (residue number corresponds to full-length herstatin) (Shamieh et al., 2004). However, while the role of the Int8 domain in HER binding is well established, its structure remains poorly understood.

Here, we characterized the structure of the isolated Int8 domain using circular dichroism (CD), nuclear magnetic resonance (NMR), and small-angle X-ray scattering (SAXS). Our results demonstrate that Int8 is intrinsically disordered with a residual helical structure. The structure prediction and modeling were consistent with experimental results. Furthermore, we show that the R371I mutant, which has a lower affinity for HER2, is prone to aggregation. Therefore, Int8 presents an interesting case, where an intrinsically disordered region (IDR) encoded by an intron possesses tumor-suppressive activity.

## 2 Materials and Methods

### 2.1 Protein Expression and Purification

The gene encoding wild-type Int8 (residues 341–419 of herstatin) (Shamieh et al., 2004) was constructed by overlap-extension PCR. To improve solubility, the N-terminus of Int8 was tagged with a 5 × Lys-tag using a Gly-Ser-Ser-Gly linker (MGKKKKKGSSG) (Kato et al., 2007). Additionally, a 6 × His-tag was linked to the C-terminus using a Ser-Ser-Gly linker (SSGHHHHHH) to facilitate protein purification. Codons were optimized for high-level expression in *Escherichia coli* using the DNAWorks server (Hoover and Lubkowski, 2002). The Int8 DNA fragment was ligated into the *Nco*I/*Bam*HI sites of the pET-15b vector (MilliporeSigma, Burlington, MA, United States). The R371I mutation was introduced according to the method of the QuikChange site-directed mutagenesis kit (Agilent Technologies, Santa Clara, CA, United States). The expression levels and solubilities of the wild-type and mutant Int8 proteins were predicted using the ESPRESSO server (Hirose and Noguchi, 2013).

Unlabeled and ^15^N-labeled Int8, as well as the unlabeled R371I mutant of Int8 were expressed in *E. coli* BL21(DE3) cells (Nippon Gene, Tokyo, Japan) in 2×YT medium (for unlabeled protein) or M9 minimal medium (for ^15^N-labeled protein) containing ampicillin (50 μg/ml). The cells were incubated at 37°C and overexpression of Int8 was induced by addition of 1 mM isopropyl β-D-1-thiogalactopyranoside at an optical density of 0.8 at 600 nm. After incubation for an additional 5 h, cells were collected, resuspended in denaturation buffer containing 20 mM Tris-HCl (pH 8.0), 6 M guanidine hydrochloride (GdnHCl), and 20 mM imidazole, and sonicated on ice for 4 min using a Branson Sonifier 250D Advanced (Branson, Danbury, CT, United States). The lysate was subsequently centrifuged at 35,140 × g for 30 min at 4°C. The supernatant was filtered through a 0.45-µm pore size membrane filter and applied to a column containing nickel-nitrilotriacetic acid agarose gel (Qiagen, Hilden, Germany). The column was washed with the denaturation buffer and wash buffer containing 20 mM Tris-HCl (pH 8.0), 500 mM NaCl, and 20 mM imidazole. Int8 was eluted using elution buffer containing 20 mM Tris-HCl (pH 8.0), 500 mM NaCl, and 0.1–1 M imidazole. For CD and light scattering measurements, the eluate was further purified by size-exclusion chromatography using a Superdex 200 pg column (Cytiva, Marlborough, MA, United States) with the buffer containing 10 mM sodium phosphate (pH 6.0) and 50 mM NaCl. For NMR and SAXS measurements, the eluate was desalted using a PD-10 column (Cytiva) in buffer containing 10 mM sodium phosphate (pH 6.0) and 50 mM NaCl. The purity of the Int8 proteins was analyzed by sodium dodecyl sulfate-polyacrylamide gel electrophoresis.

Protein concentrations were obtained using the following four methods: 1) absorption measurement at 280 nm in the presence of 6 M GdnHCl (Edelhoch, 1967; Gill and von Hippel, 1989), 2) absorption measurement at 280 nm under native conditions (Pace et al., 1995), 3) absorption measurement at 205 nm (Anthis and Clore, 2013), and 4) the Pierce BCA protein assay kit (Thermo Fisher Scientific, Waltham, MA, United States). Similar protein concentrations were obtained by all methods.

### 2.2 Circular Dichroism Measurements

Far-UV CD spectra were obtained from a J-805 spectropolarimeter (Jasco, Tokyo, Japan) at 200–250 nm using a quartz cuvette of 1 mm path length at 25°C. The temperature was controlled using a thermostat-circulating water bath. The protein concentrations of the wild type and R371I mutant of Int8 were 30 and 31 μM, respectively. The samples additionally contained 10 mM sodium phosphate (pH 6.0) and 50 mM NaCl, in the presence or absence of 4 M GdnHCl. Mean residue ellipticity (MRE) was calculated as previously described (Arai and Iwakura, 2005). The spectra in the presence of GdnHCl were measured at 211–250 nm. The helix content (*f*_H_) was estimated from the MRE at 222 nm according to the following equation (Chen et al., 1972):

$$f_{H}\;=\;-\left(\mathrm{MR}E_{\mathrm{222nm}}+\mathrm{2340}\right)\text{×}\mathrm{100}/\mathrm{30300}$$ (1)

### 2.3 Nuclear Magnetic Resonance Measurements

One-dimensional (1D) pulsed-field gradient (PFG) NMR spectra and two-dimensional (2D) ^1^H-^15^N heteronuclear single quantum coherence spectra were recorded using a Bruker Avance500 spectrometer in a buffer containing 10 mM sodium phosphate (pH 6.0), 50 mM NaCl, 10% D_2_O, and 0.2 mM sodium 3-(trimethylsilyl)-1-propanesulfonate (DSS) at 25°C. All spectra were analyzed using NMRPipe (Delaglio et al., 1995) and NMRViewJ softwares (Johnson and Blevins, 1994). The protein concentrations of the wild-type and R371I mutant of Int8 were 350 and 150 μM, respectively.

PFG NMR measurements were performed using a bipolar longitudinal eddy-current-decay pulse sequence (Wu et al., 1995; Nakamura et al., 2013; Kunihara et al., 2019). The samples contained 0.05% dioxane as a standard for molecular size analysis. The diffusion delay was 100 ms, the gradient pulse width was 6 ms, and the pulse separation was 0.6 ms. The spectral center was 4.701 ppm, and the spectral width was 8012.820 Hz. Five spectra for the peak decay of dioxane and 15 spectra for Int8 were acquired while changing the strength of the diffusion gradient (*g*) from 5.35 G/cm (5%) to 53.5 G/cm (100%). The peak intensity (*s*) is related to *g* as follows:

$$\frac{s}{s_{0}}=\exp\left(-d\cdot g^{2}\right)$$ (2)

where *s*_0_ is the peak intensity at 0% field gradient and *d* is the observed decay rate, which is proportional to the diffusion coefficient of the solute. The signal decay was fitted using Eq. 2, and the hydrodynamic radius, *R*_h_ of Int8 was calculated using the *d* value, as follows:

$$R_{h}\left(\mathrm{Int}8\right)=\frac{d\left(\mathrm{dioxane}\right)}{d\left(\mathrm{Int}8\right)}R_{h}\left(\mathrm{dioxane}\right)$$ (3)

where the *R*_h_ of dioxane is 2.12 Å (Wilkins et al., 1999).

### 2.4 Small-Angle X-Ray Scattering Measurements

SAXS measurements were conducted at beamline (BL)-10C at the Photon Factory of the High Energy Accelerator Research Organization (KEK), Tsukuba, Japan. The camera length (1,996 mm) was calibrated by the diffraction of silver behenate using FIT2D software (Hammersley, 1997). Samples were loaded into a mica-windowed cuvette with a 1 mm path length and were irradiated by a monochromatic X-ray beam (1.488 Å). Int8 concentration was 4.1 mg/ml (390 μM), and sample was centrifuged for 30 min at 4°C before measurement. The temperature in the cuvette was maintained at 25°C using a circulating water bath. Scattering images were acquired by a PILATUS3 300 KW detector (DECTRIS Ltd., Baden, Switzerland) at 10 × 1 s exposures. The scattering intensity, *I*(*Q*), was collected from 0.005 to 0.27 Å^−1^, where *Q* = 4π sin (*θ*/*λ*) (*λ*, wavelength; 2*θ*, scattering angle). Scattering of blanks (buffer alone) was measured before and/or after measurement of protein samples. Circular averaging of 2D scattering data was performed using FIT2D. Blanks were subtracted from protein solution scattering data to obtain the scattering profile of protein molecules. The subtracted scattering data were binned per four data points to increase the signal-to-noise ratio. A radius of gyration, *R*_g_, was obtained based on the Guinier approximation within the Guinier region (*R*_g_ *Q* < 1.3) (Trewhella et al., 2017):

$$\ln I\left(Q\right)=\ln I\left(0\right)-\frac{R_{\text{g}}^{2}}{3}Q^{2}$$ (4)

where *I*(0) denotes the zero-angle scattering intensity (Glatter and Kratky, 1982; Arai et al., 2007). An *R*_g_ was also estimated using the approximation of the Debye function for a random coil within the range of (*R*_g_ *Q*)^2^ < 3:

$$I{\left(Q\right)}^{-1}=a\left[1+0.359{\left(QR_{\text{g}}\right)}^{2.206}\right]$$ (5)

where *a* is the *y*-intercept of the *I*(*Q*)^−1^ versus *Q* ^2.206^ plot (Calmettes et al., 1994). The pair-distance distribution function, *P*(*r*), was calculated using GNOM software (Svergun, 1992).

The SAXS data were further analyzed using the ensemble optimization method (EOM) 2.1 (Bernado et al., 2007; Tria et al., 2015; Vallet et al., 2018). In this analysis, a pool of 10,000 random conformations based on the Int8 amino acid sequence was first generated. Then, a genetic algorithm was run 100 times to select for an ensemble of conformations that best reproduces the experimental scattering curve.

### 2.5 Size-Exclusion Chromatography and Light Scattering Measurements

Size-exclusion chromatography (SEC) measurements were performed using a high-performance liquid chromatography (HPLC) system (LP-20AP, Shimadzu, Kyoto, Japan) and a Superdex 200 increase 3.2/300 column (Cytiva). The column was pre-equilibrated with 10 mM sodium phosphate (pH 6.0) and 150 mM NaCl, and samples of the wild-type and R371I mutant of Int8 were injected onto the column. The protein concentrations were 129 and 515 μM for the wild type and 138 and 550 μM for the R371I mutant. Molecular weight of the sample was determined on the HPLC system coupled to a Viscotek TDA 305 light scattering detector (Malvern Panalytical, Malvern, United Kingdom) as described previously (Chang et al., 2020). Bovine serum albumin (66.7 kDa) was used as a standard for instrument calibration. Data analysis for estimating molecular weights was performed using OmniSEC software (Malvern Panalytical). The error in molecular weight was calculated from duplicate or triplicate measurements.

## 3 Results

### 3.1 Characterization of Int8 Structure

**FIGURE 2:**
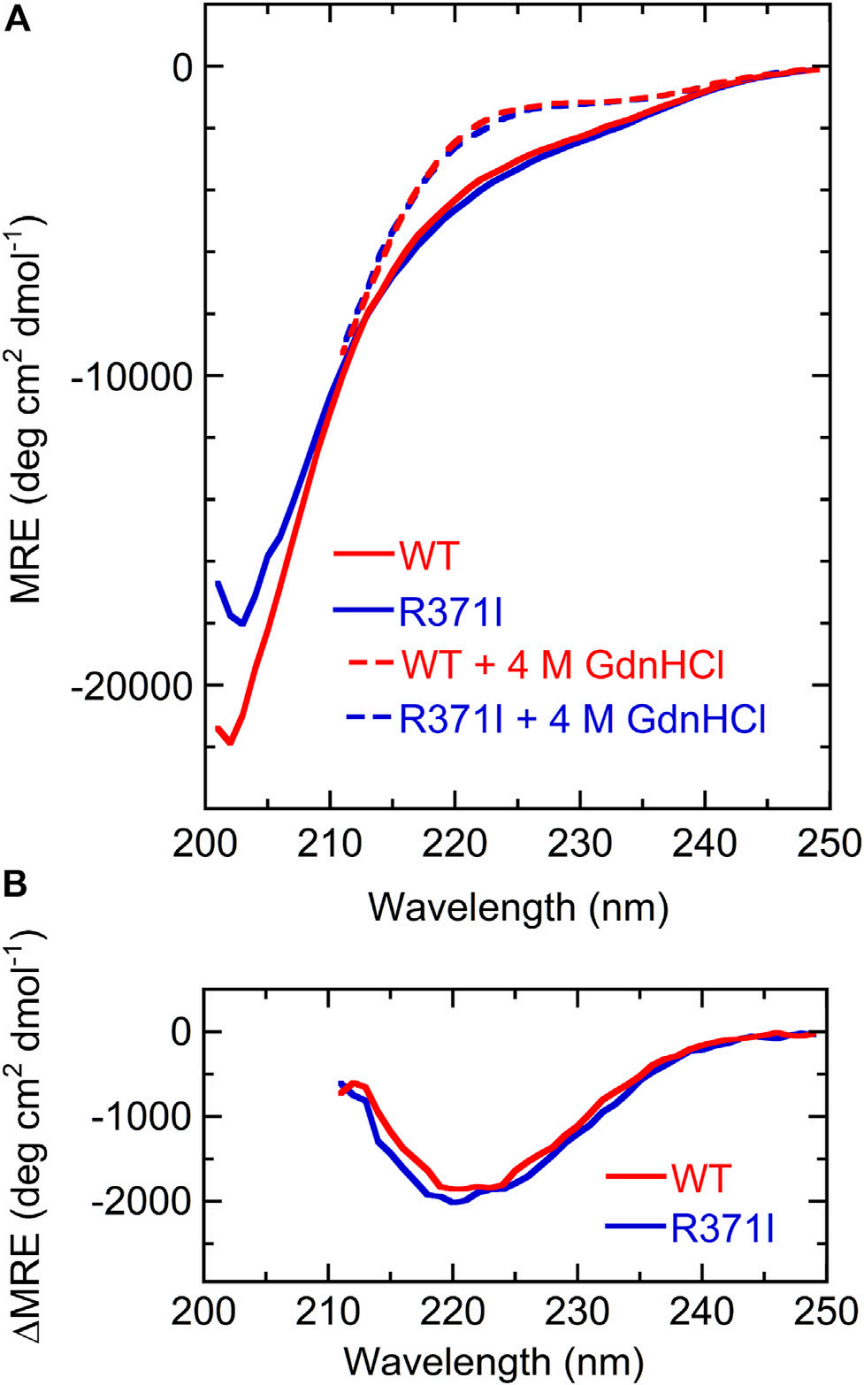
CD measurements. **(A)** Far-UV CD spectra of the wild type (red) and R371I mutant (blue) of Int8 in the absence (continuous lines) and presence (broken lines) of 4 M GdnHCl. The MRE values are shown. The spectra in the presence of 4 M GdnHCl were measured up to 211 nm due to large absorption by GdnHCl at lower wavelengths. **(B)** Difference CD spectra calculated by subtracting the CD spectrum in the presence of 4 M GdnHCl from that measured in the absence of GdnHCl.

The isolated Int8 domain containing a C-terminal 6 × His-tag and an N-terminal 5 × Lys-tag was overexpressed and purified from *E. coli*, to characterize its structure (Section 2). The structure of Int8 under native conditions was measured by CD, NMR, and SAXS. The far-UV CD spectrum of Int8 under native conditions had small intensities at ∼222 nm but had a minimum at ∼200 nm, indicating a largely disordered structure (Figure 2A). The CD intensity at ∼222 nm was reduced to almost zero after protein unfolding by the addition of 4 M GdnHCl (Figure 2A). The difference spectrum obtained by subtracting the CD spectrum in the presence of 4 M GdnHCl from that measured under native conditions showed a minimum at 222 nm, indicating the presence of an α-helical structure (Figure 2B). These findings suggest that Int8 is largely disordered under native conditions, but retains a residual helical structure. The helix content as estimated from the CD intensity at 222 nm is ∼4% (Eq. 1 in Section 2).

**FIGURE 3:**
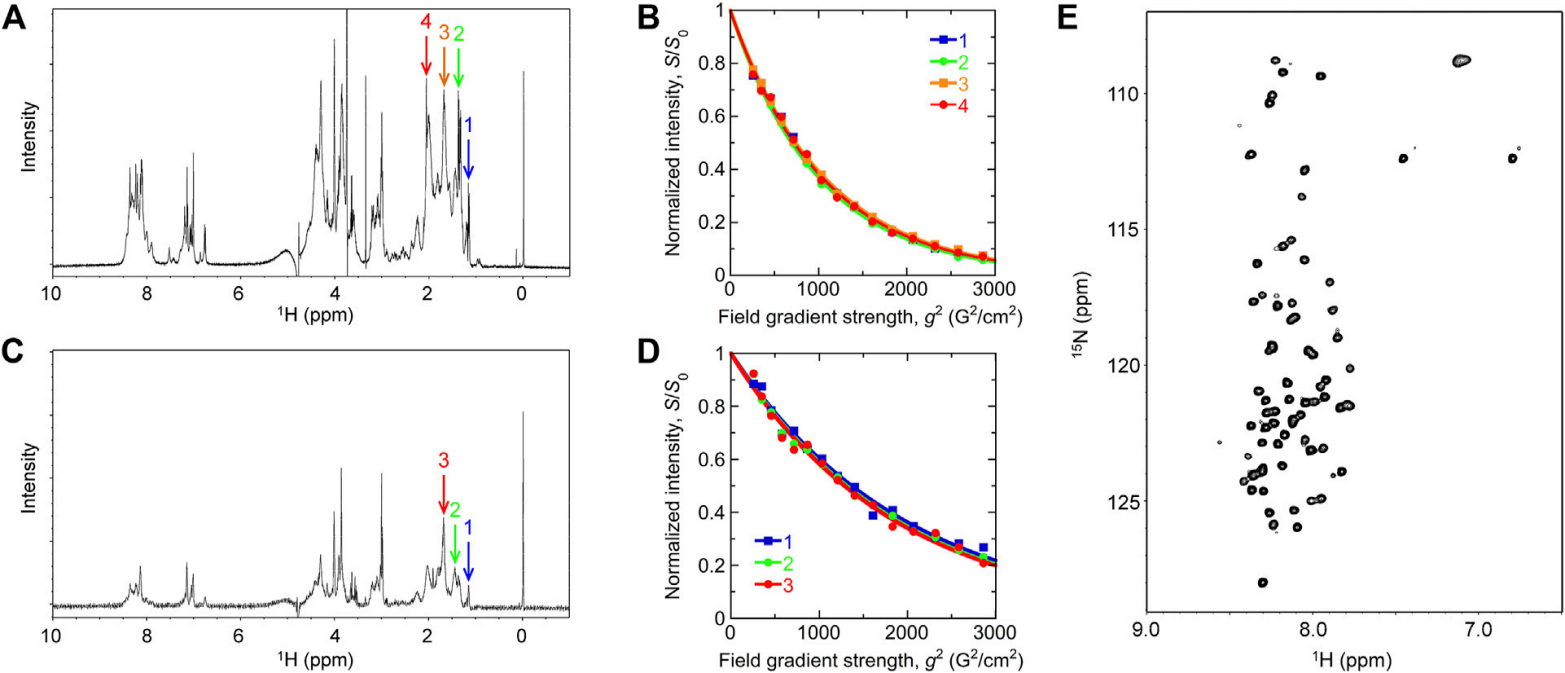
NMR measurements. **(A,C)** One-dimensional NMR spectra of the wild type **(A)** and R371I mutant **(C)** of Int8. Arrows show the peaks used for the analysis of pulsed-field gradient (PFG) NMR measurement. The DSS peak is at 0 ppm. **(B,D)** Peak intensity decay curves obtained by the PFG NMR measurement of the wild type **(B)** and R371I mutant **(D)**. **(E)** Two-dimensional ^1^H^−15^N heteronuclear single quantum coherence spectrum of the wild-type Int8.

1D and 2D NMR spectra of Int8 showed that chemical shifts of amide protons were confined in a narrow range of 7.7–8.7 ppm (Figures 3A,E). This indicates that the amide protons of Int8 are in similar environments, suggesting that Int8 is predominantly in a disordered state. PFG NMR measurements were performed to characterize the hydrodynamic radius, *R*_h_. From the 1D NMR spectra of Int8, four peaks were selected at ∼1–2 ppm (Figure 3A) and peak intensities were plotted depending on the external magnetic field gradient, *g* (Figure 3B). By fitting the decay curves (Eq. 2 in Section 2), a parameter *d* was estimated, which is proportional to the diffusion coefficient. The *d* values were found to be almost identical for the four selected peaks. Using the Einstein-Stokes equation and the *d* value of the standard substance dioxane (*R*_h_ = 2.12 Å), which was included in the NMR sample, an *R*_h_ of 33 ± 2 Å for Int8 was obtained (Eq. 3 in Section 2).

**FIGURE 4:**
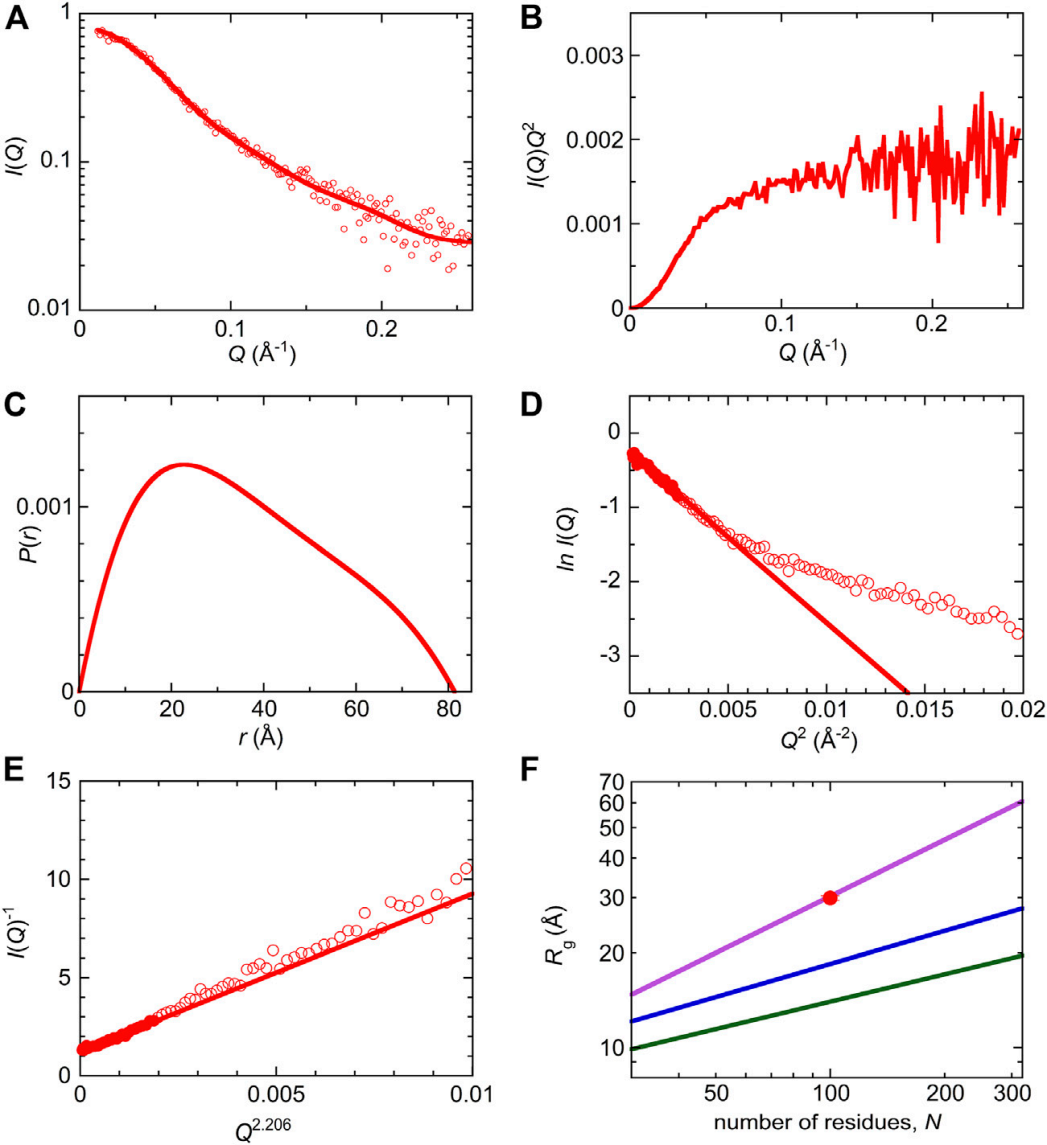
SAXS analysis of wild-type Int8. **(A)** The ln *I*(*Q*) versus *Q* plot. The continuous line was obtained by the EOM fit. The intensity is shown in an arbitrary unit. **(B)** Kratky plot. **(C)** A pair-distance distribution function, *P*(*r*). **(D)** Guinier plot. The continuous line was obtained by Guinier approximation. **(E)** The *I*(*Q*)^−1^ versus *Q* ^2.206^ plot. The continuous line was obtained by fitting to the Debye function for a random coil. **(F)** Scaling relationship for the native (green), intermediate (blue), and unfolded state (purple). The red circle shows the *R*_g_ of Int8 obtained by fitting to the Debye function.

SAXS measurements were performed to characterize the molecular size and shape of Int8 (Figure 4A). Scattering intensities, *I*(*Q*), at scattering vectors, *Q* (Å^−1^), were analyzed by a Kratky plot [*I*(*Q*)*Q* ^2^ versus *Q* plot] which provides information on the molecular shape of a protein (Glatter and Kratky, 1982; Arai et al., 2007). The presence of a peak in the plot indicates that the protein has a compact, globular shape, whereas the absence of a peak is indicative of a random coil-like disordered structure (Arai et al., 2007). The Kratky plot of Int8 lacked a peak and instead showed a plateau, indicating that Int8 has a random coil-like disordered structure under native conditions (Figure 4B). The pair-distance distribution function, *P*(*r*), calculated by Fourier transformation of *I*(*Q*), showed a peak at ∼20 Å, but the maximum distance, *D*_max_, between two atoms in Int8 was ∼80 Å (Figure 4C). It is known that compact globular particles have a symmetric bell-shaped *P*(*r*), whereas unfolded particles have an extended tail in the *P*(*r*) function (Kikhney and Svergun, 2015). Thus, the *P*(*r*) of Int8 suggests an extended structure.

Next, the SAXS data were analyzed by generating a Guinier plot (i.e., ln *I*(*Q*) versus *Q* ^2^ plot), which provides information on the molecular size of a protein, that is, the radius of gyration *R*_g_, using the Guinier approximation (Eq. 4 in Section 2). The *R*_g_ of Int8 was estimated to be 26.2 ± 0.5 Å (Figure 4D). Furthermore, the *R*_g_ estimated using the Debye function for a random coil was 29.9 ± 0.5 Å (Figure 4E; Eq. 5 in Section 2). Since the Debye function is applicable to the scattering data at wider angles (*R*_g_ *Q* < 1.73) than the Guinier approximation (*R*_g_ *Q* < 1.3), the *R*_g_ value obtained by the Debye function is less affected by interparticle interference effects (Calmettes et al., 1994). Therefore, we used an *R*_g_ of 29.9 Å for Int8. The *R*_g_ values of typical native, intermediate (molten globule), and unfolded states of globular proteins are related to the number of residues, *N*, as *R*_g_ = *R*_0_ *N* ^*ν*^, where *R*_0_ is the *R*_g_ of a single amino acid residue and *ν* is the scaling exponent (Wilkins et al., 1999; Kohn et al., 2004; Arai et al., 2007). The *R*_0_ and *ν* values are 3.68 ± 0.86 Å and 0.29 ± 0.02 for the native state (Wilkins et al., 1999), 3.68 ± 0.45 Å and 0.35 ± 0.09 for the molten globule-like folding intermediate (Arai et al., 2007), and 1.927 ± 0.271 Å and 0.598 ± 0.028 for the fully unfolded state (Kohn et al., 2004), respectively. A comparison of these values showed that the *R*_g_ of Int8 is almost the same as that of the fully unfolded state (Figure 4F).

The *R*_g_/*R*_h_ ratio is known to depend on the molecular shape of proteins. For spherical molecules (either folded proteins or disordered proteins in compact conformations) the *R*_g_/*R*_h_ ratio is $\sqrt{3/5}$ ∼ 0.78, whereas for proteins in the random-coil-like conformations it is 1.2–1.6 (Nygaard et al., 2017). The *R*_g_/*R*_h_ ratio for Int8 was 29.9/33 = 0.91, suggesting that Int8 does not have a spherical shape, consistent with the *P*(*r*) function, but is not in a fully random-coil-like state.

**FIGURE 5:**
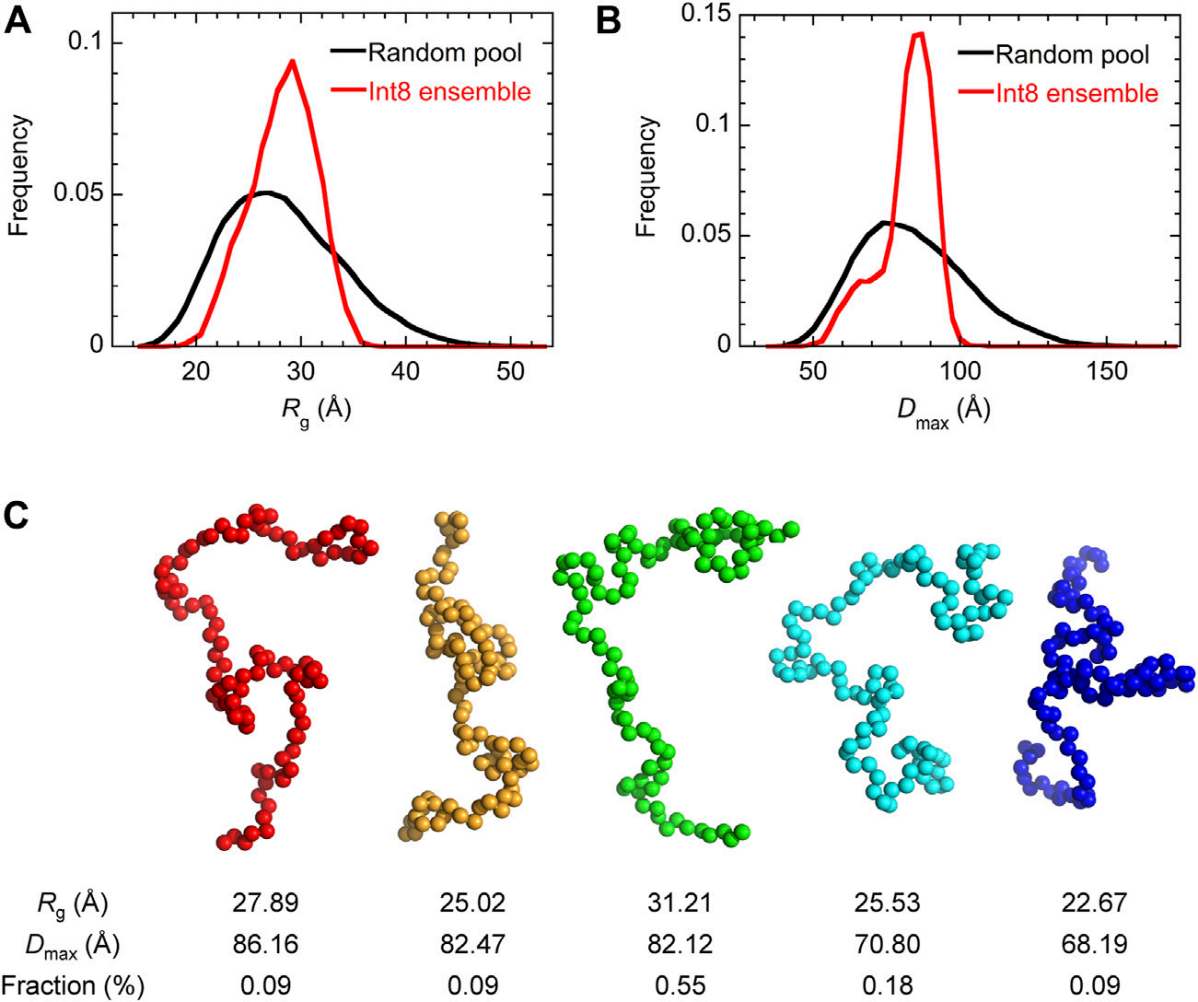
EOM analysis of the SAXS data. **(A,B)** The distribution of *R*_g_ **(A)** and *D*_max_ **(B)** for the completely random pool (black) and the ensemble of Int8 conformations (red). **(C)** Five representative conformations of the wild-type Int8 involved in the ensemble that was best fitted to the scattering curve of Int8 (Figure 4A). The *R*_g_, *D*_max_, and fraction (%) of the conformations are shown at the bottom.

Then, the ensemble of Int8 conformations that best fitted to the SAXS data was modeled by EOM (Bernado et al., 2007; Tria et al., 2015; Vallet et al., 2018). The ensemble best fitted to the scattering curve (Figure 4A; χ^2^ = 1.799) contained five representative conformations of Int8 with an averaged *R*_g_ of 28.5 Å and *D*_max_ of 79.2 Å, which are consistent with the above analysis (Figure 5). The distributions of *R*_g_ and *D*_max_ for Int8 were smaller than those for the completely random pool (Figures 5A,B). Furthermore, *R*_flex_, indicative of the flexibility of the conformational ensemble, was 71.7% for Int8, which was smaller than that for the random pool (86.2%). Thus, the SAXS data suggest that Int8 has a molecular size comparable to the fully unfolded state but is less flexible than random coils probably due to the presence of residual helical structure.

### 3.2 Structure Prediction of Int8

**FIGURE 6:**
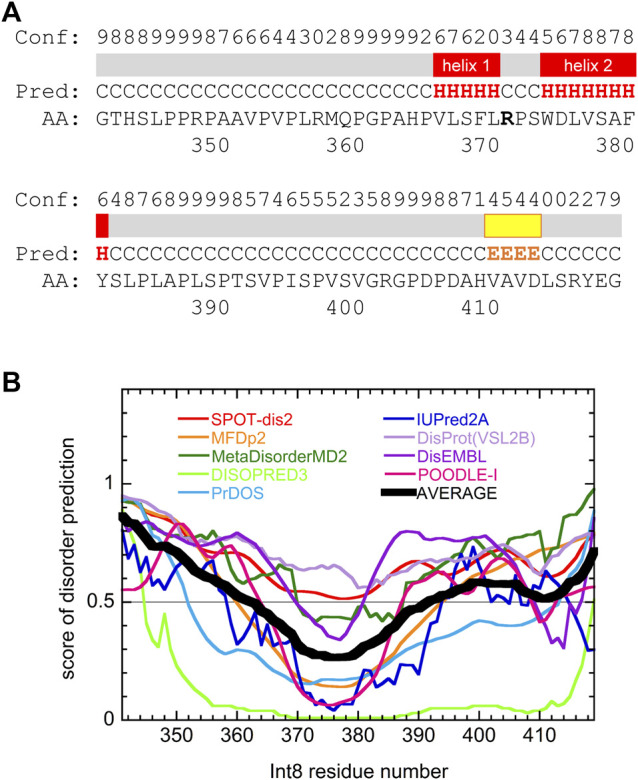
Secondary structure and disorder predictions of wild-type Int8. **(A)** Secondary structure prediction by PSIPRED. Pred indicates the predicted secondary structure (H, α-helix; E, β-sheet; and C, coil). Conf shows the confidence level of the prediction. Regions predicted to form α-helices and β-sheets are shown by red and yellow boxes, respectively. **(B)** Disorder prediction by nine different prediction servers. Black thick line shows the average of the predictions. Regions with a score larger than 0.5 are predicted to be disordered.

Secondary structure prediction of Int8 using the PSIPRED server (Buchan and Jones, 2019) showed that most regions of Int8 were in a random coil-like structure (Figure 6A). Only a small number of residues in the middle of Int8 were predicted to be organized into two α-helices [helix 1 (residues 366–370) and helix 2 (residues 374–381)] and one β-strand (residues 410–413). The AGADIR server (Muñoz and Serrano, 1994; Lacroix et al., 1998) predicted that helical propensity was low for both helices, although helix 2 (residues 373–382) had a higher helical propensity than helix 1 (residues 365–369) (Supplementary Figure S1). These predictions are consistent with the CD measurements that showed the presence of only a small amount of helical structure in Int8.

Disorder prediction was performed using nine prediction servers, namely SPOT-Disorder2 (Hanson et al., 2019), MFDp2 (Mizianty et al., 2013), MetaDisorderMD2 (Kozlowski and Bujnicki, 2012), DISOPRED (Jones and Cozzetto, 2015), PrDOS (Ishida and Kinoshita, 2007), IUPred2A (Meszaros et al., 2018), DisProt(VSL2B) (Obradovic et al., 2005), DisEMBL (Linding et al., 2003), and POODLE-I (Shimizu, 2014). Although the results obtained differed between individual prediction servers, many predicted that most regions of Int8, particularly in the N- and C-terminal regions were intrinsically disordered (Figure 6B), which was consistent with the secondary structure predictions. The disordered nature of Int8 is probably due to the presence of many proline and serine residues between residues 346–365 and 384–406. Thus, theoretical predictions suggest that Int8 is intrinsically disordered with a residual helical structure, consistent with our experimental results (Figures 2–5).

### 3.3 Structure of the R371I Mutant

The structure of the R371I mutant of Int8, which does not bind HER2 (Shamieh et al., 2004), was predicted by PSIPRED and the disorder prediction servers to be similar to that of the wild-type Int8 (Supplementary Figure S2). The AGADIR server also predicted that the helical propensity of the R371I mutant is almost the same as that of the wild-type Int8 (Supplementary Figure S1).

The R371I mutant of Int8 was overexpressed in *E. coli* and purified. The CD spectrum of the R371I mutant of Int8 was found to be similar to that of wild-type Int8 (Figure 2A). The helix content of the mutant was estimated to be ∼5% (Eq. 1 in Section 2). The difference CD spectrum between those measured in the absence and presence of 4 M GdnHCl had a negative peak at ∼222 nm in the R371I mutant (Figure 2B). These results indicate that the mutation little affected the helix content of Int8, which is consistent with the theoretical predictions (Supplementary Figure S1).

The 1D NMR spectrum of the R371I mutant showed that peaks for amide protons are confined in a narrow range of 7.7–8.7 ppm (Figure 3C), indicating disordered structures. The molecular size of the R371I mutant of Int8 was estimated by PFG NMR measurement. Three peaks at ∼1–2 ppm in the 1D NMR spectra of the mutant were used for the analysis of peak intensity decays (Figures 3C,D). The *R*_h_ for the R371I mutant was 72 ± 2 Å. This value is more than two-fold larger than that of wild-type Int8, indicating the formation of soluble aggregates in the mutant. The aggregation was observed despite the use of a lower protein concentration for the R371I mutant (150 μM) than for the wild-type Int8 (350 μM). Therefore, these results indicate that the R371I mutant of Int8 is prone to aggregation.

**FIGURE 7:**
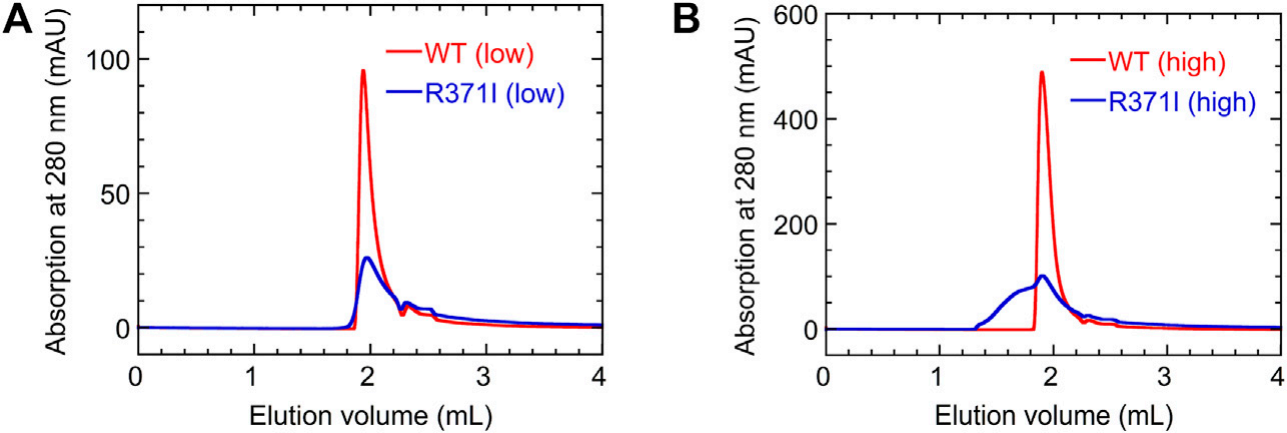
SEC measurements. **(A,B)** Elution profiles of the wild type (red) and R371I mutant (blue) of Int8 measured at low (∼100 μM) **(A)** and high (∼500 μM) protein concentrations **(B)**.

To further investigate the aggregation propensity of the mutant in more detail, we performed SEC measurements for the wild type and R371I mutant of Int8 (Figure 7). The elution profile of the wild type showed a single peak without aggregates at both low (∼100 μM) and high protein concentrations (∼500 μM). The molecular weights estimated by static right-angle light scattering were 12.6 (±0.1) kDa and 13.2 (±0.1) kDa for the low and high concentration samples of the wild-type Int8, respectively (Supplementary Figure S3), both of which are close to the value expected for an Int8 monomer (10.5 kDa). In contrast, a large amount of aggregates were observed at a high concentration (∼500 μM) of the R371I mutant (Figure 7B) with a molecular weight of 20.3 (±0.5) kDa at the elution peak (Supplementary Figure S3). At a low protein concentration (∼100 μM), the formation of aggregates was suppressed (Figure 7A), and the molecular weight at the elution peak was of 11.3 (±0.6) kDa, which is close to the value for an Int8 monomer (Supplementary Figure S3). However, the mutant started to elute earlier than the wild type, indicating the presence of slight aggregates (Figure 7A). The large *R*_h_ of the mutant determined by the PFG NMR measurements at 150 μM may correspond to the molecular size of these aggregates. Taken together, these results clearly demonstrate that the R371I mutant of Int8 is prone to aggregation.

### 3.4 Structure Modeling of Int8

**FIGURE 8:**
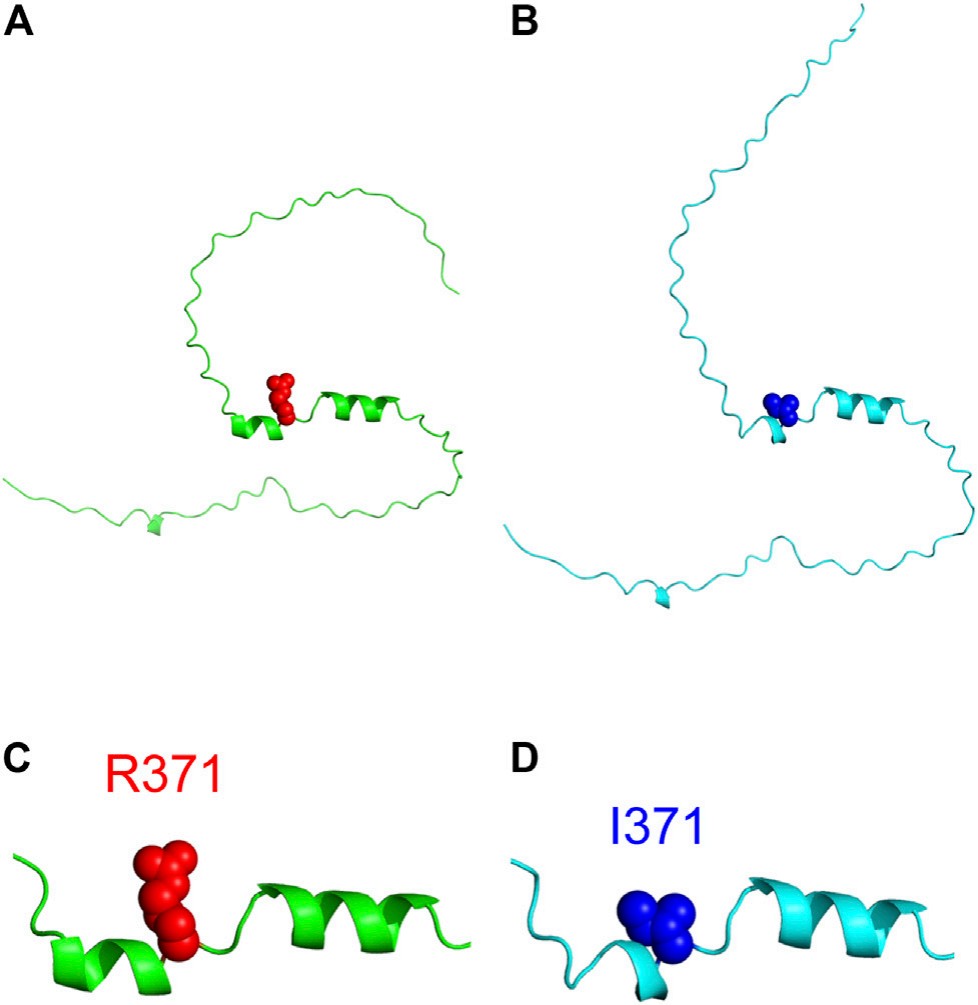
Structure modeling of Int8. **(A,B)** Overall structures of the wild type **(A)** and R371I mutant **(B)** of Int8 predicted by AlphaFold2. **(C,D)** Expanded views of the helical regions of the wild type **(C)** and R371I mutant **(D)**. R371 and I371 are shown by red and blue balls, respectively. The Figures were drawn using the PyMOL Molecular Graphics System, Version 2.4.0 Schrödinger, LLC.

Three-dimensional structure prediction of the wild type and R371I mutant of Int8 was performed using the standalone version of AlphaFold2 (Jumper et al., 2021). The predicted structure of wild-type Int8 was largely disordered, but had two α-helices at residues 365–370 and 374–382 corresponding to helices 1 and 2, respectively (Figures 8A,C). The predicted structure of the R371I mutant was similar to that of the wild type (Figure 8B), except that helix 1 (residues 368–370) was shorter (Figure 8D), which may be consistent with the slightly reduced helical propensity predicted by AGADIR for the mutant (Supplementary Figure S1).

## 4 Discussion

In this study, we characterized the structure of Int8, the intron 8-encoded domain of herstatin. Since Int8 binds and interferes with the homo- and hetero-dimerization of HER1, HER2, and HER4, it serves as an intrinsic inhibitor of the HER family proteins. Structure prediction and experimental characterization by CD, NMR, and SAXS indicated that Int8 is largely disordered, but retains a residual helical structure. Moreover, it had a molecular size similar to that of the fully unfolded state, although the conformational ensemble was less flexible than random coils. These results clearly indicate that Int8 is intrinsically disordered. To our knowledge, this is the first report of an IDR encoded by an intron. Further, the structure of the Int8 domain might be classified as a pre-molten globule state, which has an expanded overall structure but with unstable secondary structure (Ptitsyn, 1995; Uversky, 2002a; b; Tcherkasskaya et al., 2003). Pre-molten globule-like structures have also been reported for other intrinsically disordered proteins (IDPs) (Ptitsyn, 1995; Uversky, 2002a; b; Tcherkasskaya et al., 2003; Kunihara et al., 2019). Since Int8 is known to have tumor-suppressive activity (Lv et al., 2012), it is an interesting example of an intron-encoded IDR that may function as an antitumor drug.

Many IDPs exhibit coupled folding and binding behaviors, where binding is accompanied by folding (Arai, 2018). In many cases, the residues involved in coupled folding and binding have a propensity to form a helical structure (Mohan et al., 2006). Therefore, the residual helical structure present in Int8 may serve as the putative HER-binding site of Int8.

Previous reports have shown that intrinsic disorder is involved in protein-protein interactions mediated by HER proteins. The intracellular kinase domain of HER1 contains an IDR at the dimerization interface, which reorganizes into an ordered structure upon dimerization (Shan et al., 2012). Furthermore, HER1 dimerization is facilitated by cancer-related mutations that suppress local disorders at the dimerization interface (Shan et al., 2012). Therefore, intrinsic disorder regulates the dimerization of HER proteins. Thus, the use of intrinsic disorder in the interaction between herstatin and HER proteins is not exceptional. Rather, IDRs may play an important role in regulating protein-protein interactions related to cell proliferation (Uversky et al., 2008).

## Data Availability

The original contributions presented in the study are included in the article/Supplementary Material, further inquiries can be directed to the corresponding author.

## Abbreviations

1D, one-dimensional; 2D, two-dimensional; CD, circular dichroism; *D*_max_, maximum distance; DSS, sodium 3-(trimethylsilyl)-1-propanesulfonate; ECD, extracellular domain; EOM, ensemble optimization method; GdnHCl, guanidine hydrochloride; HER, human epidermal growth factor receptor; HPLC, high-performance liquid chromatography; *I*(0), zero-angle scattering intensity; IDP, intrinsically disordered protein; IDR, intrinsically disordered region; *I*(*Q*), scattering intensity; NMR, nuclear magnetic resonance; PFG, pulsed-field gradient; *P*(*r*), pair-distance distribution function; *Q*, scattering vector; *R*_g_, radius of gyration; *R*_h_, hydrodynamic radius; SAXS, small-angle X-ray scattering; SEC, size-exclusion chromatography; UV, ultraviolet.
