# Supplementary material for: Intron-Encoded Domain of Herstatin, An Autoinhibitor of Human Epidermal Growth Factor Receptors, Is Intrinsically Disordered
Source: Front Mol Biosci. 2022 May 2;9:862910. doi: 10.3389/fmolb.2022.862910 (PMC9100580; doi:10.3389/fmolb.2022.862910)
Supplement: Supplementary file 1 [file DataSheet1.PDF]

# *Supplementary Material*

## Supplementary Figures

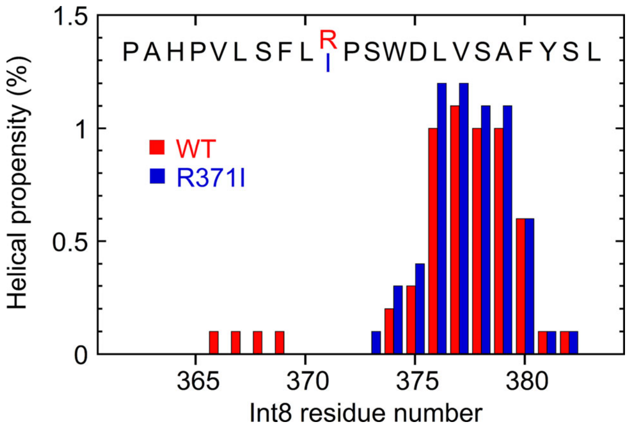


**Supplementary Figure S1.** Prediction of helical propensity by AGADIR for the wild type and R371I mutant of Int8 at residues 361–384. pH and temperature were set to 6.0 and 298 K, respectively, to match them with the experimental conditions. The amino acid sequences are shown at the top.

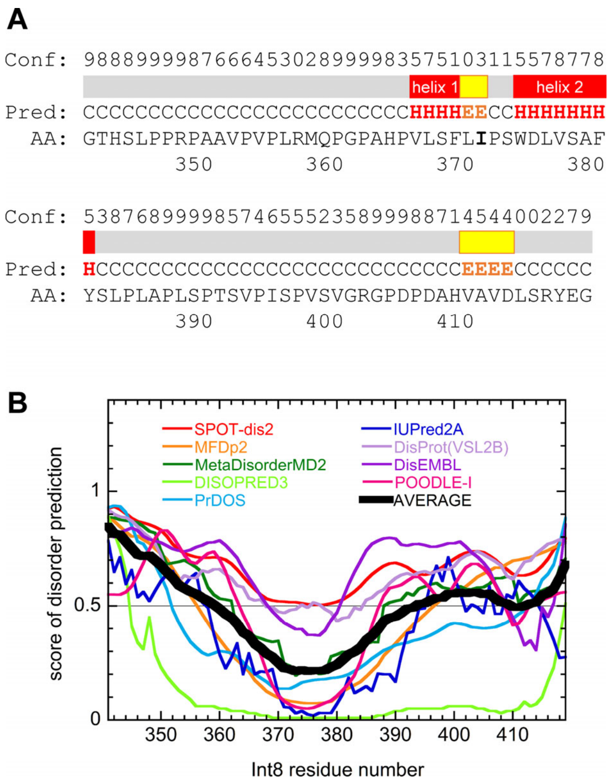


**Supplementary Figure S2.** Secondary structure and disorder predictions of the R371I mutant of Int8. **(A)** Secondary structure prediction by PSIPRED. **(B)** Disorder prediction by nine different prediction servers. Details are the same as described in Figure 6.

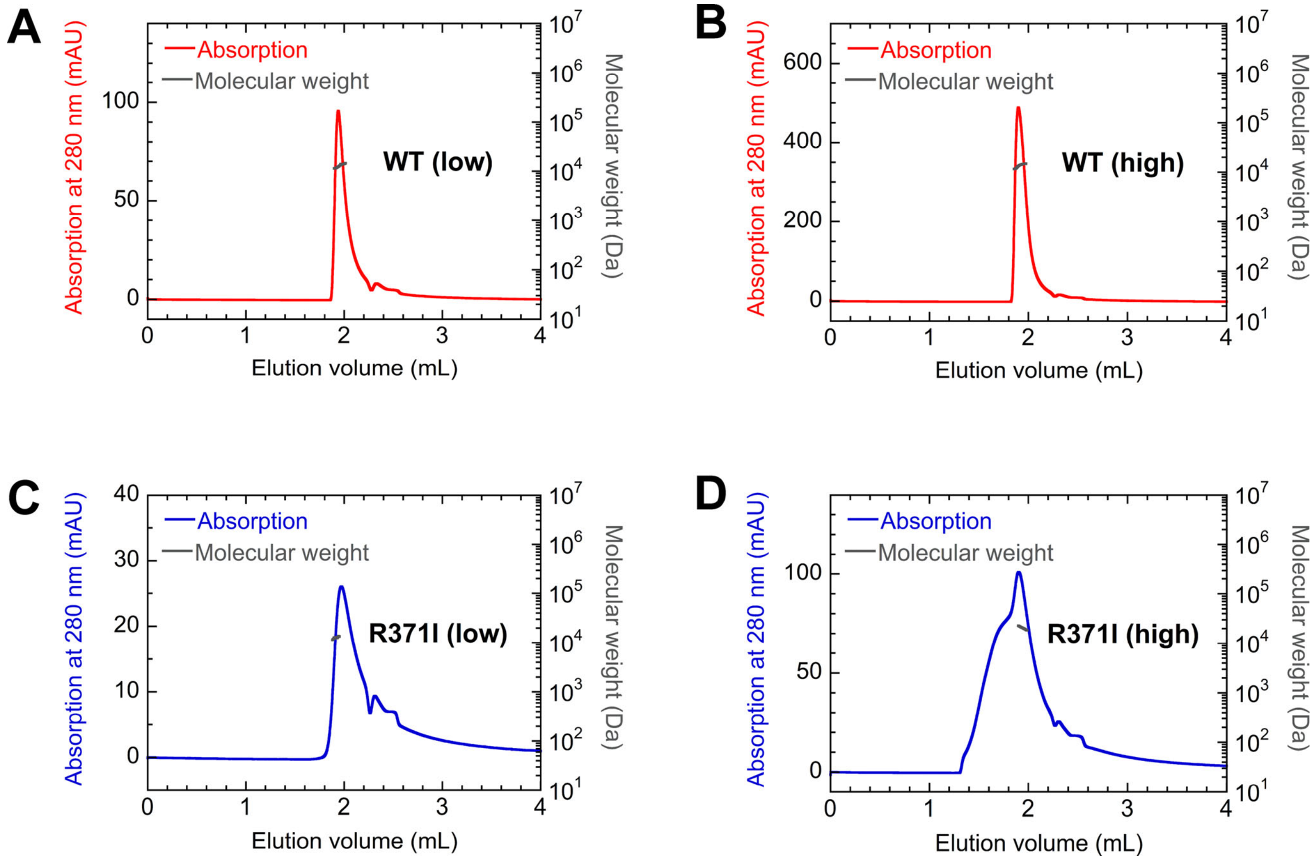


**Supplementary Figure S3.** Molecular weights of the wild type and R371I mutant of Int8 measured by static right-angle light scattering. Size-exclusion chromatography elution profiles of the wild type (red) and R371I mutant of Int8 (blue) and molecular weights (gray) are shown. **(A, B)** The molecular weights of the wild-type Int8 at low (~100 μM) and high (~500 μM) protein concentrations were 12.6 (± 0.1) kDa and 13.2 (± 0.1) kDa, respectively. **(C, D)** The molecular weights of the R371I mutant of Int8 at low (~100 μM) and high (~500 μM) protein concentrations were 11.3 (± 0.6) kDa and 20.3 (± 0.5) kDa, respectively.
